# Embedding responsibility in intelligent systems: from AI ethics to responsible AI ecosystems

**DOI:** 10.1038/s41598-023-34622-w

**Published:** 2023-05-18

**Authors:** Bernd Carsten Stahl

**Affiliations:** 1 School of Computer Science, Jubilee Campus, Wollaton Road, Nottingham, NG8 1BB UK; 2 Centre for Computing and Social Responsibility, De Montfort University, The Gateway, Leicester, LE19BH UK

**Keywords:** Computer science, Information technology

## Abstract

Intelligent systems that are capable of making autonomous decisions based on input from their environment have great potential to do good, but they also raise significant social and ethical concerns. The discourse on ethics and artificial intelligence (AI) has covered these concerns in depth and developed an array of possible ways of addressing them. This article argues that a shortcoming of this discourse is that it concentrates on specific issues and their mitigation but neglects the nature of intelligent systems as socio-technical systems of systems that are often described as ecosystems. Building on the discussion of ethics and AI, the article suggests that it would be beneficial to come to an understanding of what would constitute responsible AI ecosystems. By introducing the concept of meta-responsibility or higher-level responsibility, the article proposes characteristics that an ecosystem would have to fulfil, in order to be considered a responsible ecosystem. This perspective is theoretically interesting because it extends the current AI ethics discourse. It furthermore offers a novel perspective for researchers and developers of intelligent system and helps them reflect on the way they relate to ethical issues.

## Introduction

Intelligent systems that are capable of making autonomous decisions based on input from their environment have great potential to do good, but they also raise significant social and ethical concerns. The debate around the ethics of AI has mushroomed in recent years, covering a range of topics from biases in algorithmic decision making, fairness and reliability to broader societal concerns such as the economic and political dominance of large tech companies. The possibility of truly human-like AI and discussions of its potential moral status has been a topic of discussion for decades and has received renewed attention in light of recent rapid technical progress.

This article looks at the ethics of AI debate and focuses on one particular aspect that calls for further attention, namely the question: where is (moral) responsibility for intelligent systems located? The current AI debate uses numerous different terms, such as trustworthiness, safety, reliability and human-centredness which have strong ethical connotations and refer to specific aspects of ethics. Ethics can be understood as the field that investigates the difference between good and bad, right and wrong and the theoretical bases on which such distinctions can be drawn. Responsibility, or more specifically moral responsibility^1^, refers to the question of who or what is answerable for a state of affairs that is ethically relevant. The term ‘responsible intelligent systems’ can thus be used to refer to the question of who is answerable for ethically relevant uses or outcomes of intelligent systems.

The answer to this question is well-discussed and depends on the underlying technology, its application, social context, and other factors. Prominent candidates for the answer to the question of who or what is responsible for intelligent systems include the developer(s), user(s) in various functions, e.g. as consumers but also as co-designers, individual or corporate owner(s), societal actors including regulators and legislators, or even the technical system itself.

The AI ethics debate demonstrates that none of these potential actors provide convincing answers to the question of where responsibility for intelligent systems is located. By drawing on the AI ethics debate and the well-established discourse on moral responsibility, this article suggests that a different way of conceptualising responsibility in the context of intelligent systems is called for. It is well-established that intelligent systems are examples of complex socio-technical systems. These are typically nested and build on one another, so that one can understand them as systems of systems. The complexity of the relationship of such systems has given rise to the use of the metaphor of ecosystems to describe the AI landscape. This article explores what the understanding of intelligent systems as ecosystems means for questions of ethics and responsibility. It suggests that responsibility considerations need to be integrated into the understanding of the ecosystem. The article then unpacks the theoretical and practical implications that such a position has for our understanding of the location responsibility for intelligent systems. It proposes that the application of the idea of a meta-responsibility is called for, i.e. that we need to consider which responsibility ascriptions are required to render the overall ecosystem responsible.

This argument is of interest to several stakeholder groups. It is relevant to scholars pursuing questions of ethics of AI from perspectives of philosophy or social sciences. It is, however, also important for scientific and technical experts who design and develop intelligent systems. These experts are generally aware of the ethics-related discussion of their work, but often find it difficult to give practical responses to the question of moral responsibility for the results of their efforts. Embedding responsibility into ecosystems of intelligent systems provides them with an avenue of reflecting on ethical questions more holistically and thereby finding options that are more likely to be socially acceptable.

In order to develop this argument, the article proceeds as follows. It starts with a brief overview of AI ethics which is followed by a discussion of the concept of responsibility in the current AI ethics debate. Based on the limitations of dominant models of responsibility, the next section introduces the concept of ecosystems of intelligent systems and proposes the idea that these call for a higher level of responsibility or ‘meta-responsibility’. The conclusion spells out the theoretical and practical implications arising from this argument.

## AI ethics

The discussion of ethical, social and related questions in this article focuses on the concept of AI. AI is a much-used term that lacks a commonly accepted definition. In this article we follow Hall and Pesenti^2^ in seeing AI as an umbrella term that covers a set of complementary techniques that have developed from statistics, computer science and cognitive psychology. More important than an exact definition and enumeration of the underlying technologies and approaches is the recognition that AI is the core enabler of intelligent systems. Where such systems interact with their environment and act autonomously, i.e., perform functions without explicit human instructions, this is based on their integrated AI-enabled capabilities. It is therefore appropriate to base this article on the AI ethics discourse.

Ethical concerns about AI can be traced back to the early stages of digital computing^3–5^. There has been a significant amount of research on the topic. However, the interest in these ethical concerns has shot up in parallel to the scientific progress and practical impact of AI in the last decade. This recent success of AI is typically explained by the availability of large data sets, software tools and computing power which enabled established AI approaches such as deep learning to successfully solve practical problems^6^.

### Ethical issues and responses

It is not possible to do justice to all nuances of the mushrooming AI ethics debate in this short article. At the same time, it is important to understand the limitations of this debate. Therefore, this article provides a brief overview of some of the most widely discussed ethical issues and suggestions on how these may be addressed^7^.

Many of the ethical concerns that are discussed in the AI ethics discourse are not confined to AI and have their roots in digital technologies more broadly. However, they are often exacerbated by AI. The present focus on machine learning in the AI ethics debate furthermore leads to a focus on ethical issues that are linked to some of the theoretical and practical implications of machine learning, notably its requirement for large datasets for training and validation purposes, the opacity of many algorithms and techniques and the significant computational needs of machine learning.

Some of the ethical issues that are most prominently discussed can be traced back to these features of machine learning. This is true for the probably most widely discussed issue of fairness^8,9^ which appears to be threatened because autonomous systems can make decisions or categorise individuals on the basis of inappropriate criteria. Prominent examples are those of discrimination against individuals^10^ on the basis of implicit biases hidden in training data, leading to discrimination on the basis of race, gender, age and other protected characteristics. These concerns are exacerbated because of a lack of transparency of how systems arrive at decisions or categorisations^11,12^. This lack of transparency engenders a lack of accountability^13^ rendering the finding of resolutions more difficult. Another example of AI exacerbating established ethical concerns is that of privacy and data protection^14,15^. The need for access to large datasets and the ability to learn from those and combine them means that AI can pose novel threats to data protection^16^, for example by collecting new data types or facilitating automated surveillance.

It has been suggested that ethical concerns about AI can be categorised using a temporal dimension^17,18^. When using this categorisation, the concerns around fairness, discrimination, privacy, security, reliability etc. fall in the category of near-term concerns^19^. In addition to these, there are long-term concerns that either materialise due to the established use of these technologies or are expected because of possible future technical developments. These include questions concerning the socio-economic consequences of AI use^20^ such as the future of work^21,22^ including possible AI-based unemployment as well as broader questions around the justice of distribution of AI-derived economic gain^23,24^. Similar longer-term issues include the impact of AI on policy and democratic processes, for example where economic power but also technical expertise are used to influence democratic decisions^25,26^. The military use of AI, potentially automating the killing of humans^27,28^ or environmental impacts are further longer-term concerns. In addition, there are discussions about the nature of AI, whether it can and will become more human-like^29^, replace humans^30^, become super-intelligent^31^ or conscious and become a moral subject in its own right. While these latter points are strongly contested^32^, they are worth considering because they have a high level of visibility in the public imagination and media discourse.

This brief and non-comprehensive overview of the ethics of AI demonstrates the breadth and richness of the discussion. It says very little about how these various concerns can be addressed. This will be looked at in the next section on “Responsible AI”.

### Responsible AI

**Figure 1:**
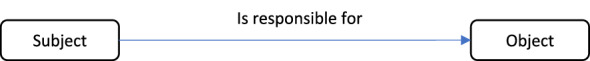
Simple responsibility relationship.

This article uses the term ‘responsible AI’^33^ to denote the attempt to find practical ways of dealing with the various ethical and related issues. It is based on a long discussion of the concept of responsibility in law, social sciences and moral philosophy^1^. The root of the term lies in the response, in the ability and willingness to answer. Responsibility is a relational concept, which is often described as linking a subject to an object, i.e., determining who is responsible and what they are responsible for. A prominent example of this is criminal legal responsibility^34^ where the subject is the offender and the object is the crime they committed. The following Fig. 1 represents this simple relationship.

This example of legal responsibility also shows that responsibility relationships are always more complex than a simple link between subject and object. They often include an authority (e.g. judge or jury in criminal responsibility), normally require a normative basis (e.g. criminal law) and typically have consequences in the form of sanctions or rewards. A more detailed analysis shows that responsibility relationships normally have many components that influence how responsibility is perceived, allocated and realised^35^.

**Figure 2:**
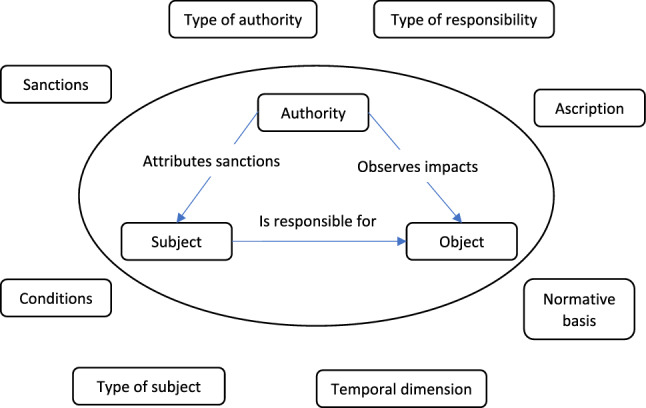
Components and influence factors of responsibility relationships.

Figure 2 represents a more detailed view of responsibility. The three components within the ellipse are those just outlined, i.e. the subject that is responsible, the object that the subject is responsible for and the authority that determines and enforces the practical consequences of the responsibility relationship. The concepts surrounding the core responsibility relationship influence the social reality of this ascription. One key concept relates to the type of responsibility. So far, we have alluded mostly to moral responsibility and legal responsibility but one can have role responsibilities or others. All of these have a moral component, but they may look very different, depending on which type is in the foreground.

Other aspects that are typically important and determine what a responsibility relationship looks like in practice are the sanctions (punishments, rewards), the type of authority (e.g. judge, personal conscience, social opinion), the mechanism of ascription (reflexive (self-ascription) or transitive (ascription to another)), the temporal dimension (prospective or retrospective), the type of subject (e.g. individual human being, organisation, technical artefact) or the conditions that a subject is deemed to have to fulfil (e.g. rationality, ability to react to sanctions). It is easy to see that these concepts are interdependent to some degree but still leave space for numerous permutations.

In the context of intelligent systems, the question of responsibility can thus be translated into the question: who or what is answerable for the design and use of such systems and their consequences. This implies follow-up questions such as how is responsibility allocated and by whom or what are the consequences of the attribution of responsibility? One important aspect of this debate is that it normally purports to be practical. While ethical issues of AI can be explored in a purely descriptive and detached sense, speaking of responsible AI conveys the impression of practical consequences.

There is, indeed, no shortage of proposals suggesting ways of addressing the various ethical issues. Many of these aim to provide guidance to AI experts on how to ensure that ethical issues do not arise or can be mitigated. This includes work on opening up AI to critical scrutiny, for example by rendering it explainable^36,37^ or including mechanisms that allow a forensic examination of AI, where things go wrong^38^. One approach to ensuring responsibility is to integrate AI design and development in existing mechanisms aimed to ensure responsibility^39^, such as risk management frameworks^40,41^. Such governance structures can point to a rapidly growing number of tools that aim to deal with various issues^42^. A closely related set of ideas offer suggestions for development methodologies that incorporate ethical sensitivity^43,44^. Such tools and methodologies can draw on and be inspired by a large number of ethics principles and frameworks^45,46^ and find their expression in ethics codes^47^ which may be part of professional guidance^48^ and include process of standardisation^49,50^ and certification^51,52^.

In addition to these approaches to promote responsibility in the design, development and use of AI, there are a number of initiatives that aim to further integrate AI into existing mechanisms of ascribing responsibility. This includes suggestions such as the further integration of AI ethics into organisational governance^53^ which can cover areas such as data governance^54^ or AI impact assessments^55,56^. On the political level one can observe movements to apply existing regulatory mechanisms to AI, for example by highlighting AI’s impact on human rights and tailoring human rights approaches to AI^11,57^. In addition one can observe moves towards specific legislation for AI such as the EU’s AI Act^58^, the US’s proposal for an AI Bill of Rights^59^ or the UK’s public interest data bill^54^. Such a broader regulatory framework could also include new national^60^ or international^61^ regulatory bodies. These legislative options are being considered in various jurisdictions. The EU's AI Act may be the most advanced in terms of a legislative agenda with a White Paper^62^ having been published in 2020 and the proposed Regulation^58^ published and debated since 2021. The various regulatory and legislative agendas are well described on the European level^63^ but various other proposals exist in other parts of the world^64^.

This brief overview of some of the more prominent proposals to mitigate ethical concerns about AI shows that responsible AI currently comprises a complex arrangement of individual, collective and institutional responsibilities. Many of these are well-established whereas others are novel and emerging. A key challenge is that most of these responsibilities are focused on the individual human being as subject. There has been a long-standing debate about collective responsibility^65^ with a particular focus on corporate responsibility^66^. The current AI ethics discourse reflects this to some degree, but it still focuses on individual subjects and sometimes corporate subjects being responsible for specific consequences of AI that are deemed to be ethically problematic. This article questions whether this conceptual basis of responsible AI is sufficient to come to a satisfactory account of responsible AI.

## Ethics of ecosystems

The argument put forward in this article is based on the recognition that intelligent systems are classical examples of socio-technical systems^67^. This means that they consist of an assemblage of heterogenous components including individual humans, technological artefacts, and social structures. Ethical issues arise when these socio-technical systems interact with their environment. But it is rarely possible to draw a clear line between one particular component of the system and a clearly defined outcome. As is typically the case for systems, the overall system is greater than the sum of its parts. And to complicate matters even further, any individual intelligent system (e.g. a fraud detection system in an insurance or an autonomous vehicle) is embedded in and forms part of a broader set of technical and social systems.

### Ecosystems of socio-technical systems

Based on empirical observations of ten existing sets of intelligent systems^68^ and exploratory work on emerging systems^12^, it has been suggested that it would therefore be useful to apply the metaphor of an ecosystem to understand the dynamics of intelligent systems^69,70^. Ecosystems consist of different actors which exist in a shared space and frame of reference. These actors may compete or collaborate. Ecosystems may flourish and grow or wither and die. Ecosystems can be nested, i.e. they can overlap or include sub-systems. Interventions in ecosystems can have unexpected positive or negative consequences. Briefly, the metaphor of an ecosystem describes many of the aspects of intelligent systems and has therefore been adopted by several high-level policy-oriented interventions such as the EU’s AI High Level Expert Group^71^, the OECD^72^, UNESCO^55^ and the UK government^73^.

The adoption of the ecosystem metaphor has the advantage of providing a strong conceptual basis for an improved understanding of the social reality of intelligent systems. It can draw from a significant discourse in fields like innovation studies that has developed conceptual and empirical tools for understanding and shaping ecosystems^74,75^. This may explain the wide-spread adoption of the terminology in particular in the policy-oriented discourse. However, from the perspective of this article, the use of the ecosystem metaphor raises challenges for dealing with ethical concerns. The application of the idea of moral responsibility to ecosystems is problematic. Theoretical descriptions of subjects of responsibility have identified a number of specific requirements that such a subject needs to fulfil, in order to be ascribed responsibility. These requirements include having awareness, an ability to understand their position, an ability to react to external stimuli (such as reward or punishment), agency, rationality, and the power to effect change^76^. These conditions are already difficult to ascertain in the traditional case where the subject of responsibility is an individual rational human being. They point to some of the most difficult philosophical questions such as the possibility of free will and freedom of action or the link between cognition and action. It is unlikely that a sociotechnical ecosystem can be reasonably portrayed as a subject of responsibility in the traditional sense.

This leaves us with a conundrum. On the one hand, it can be argued that it makes sense to view any intelligent system as an ecosystem, or maybe better as an ecosystem of ecosystems, which consists of many different systems defined by particular artefacts, locations or systems members. This ecosystem view explains the social and ethical consequences that can be observed better than a view that focuses on particular technical artefacts, organisations or individuals. On the other hand, an ecosystem cannot serve as a subject of responsibility, cannot be held responsible for the ethical issues that arise within it. A responsible ecosystem of intelligent systems thus needs to be conceptualised differently.

This raises the question: what would constitute a responsible ecosystem of intelligent systems? We have just ruled out the answer that this would be an ecosystem that is deemed to be directly responsible for the ethical and social consequences of its component parts. However, there is a different way of thinking about responsible ecosystems. If we return to the suggestion offered above that the use of the term ‘responsible intelligent system’ refer to the question of who is answerable for ethically relevant uses or outcomes of intelligent systems, then a responsible ecosystem is one that provides an answer to the question of who is answerable for the uses or consequences of the action of the system. This does not require that the ecosystem itself acts as an agent comparable to a human being, but it calls for structures that support and confirm the ability to answer to ethical concerns.

This view of responsible ecosystems has the advantage that it is open to all of the various mitigation approaches outlined earlier. It does not negate any of the existing responsibility relationships. The individual programmer is still responsible for the quality of the code they write, the company is still responsible for the consequences of the intended use of the systems it employs. The benefit of the ecosystems perspective is that it allows the recognition of the complexity of the network of existing responsibilities. A responsible ecosystem would then be one where the existing complexity of responsibility relationships is recognised and retained and which allows for the intervention in the network of responsibilities to ensure that they support each other, create synergies and collectively promote consequences that are beneficial, acceptable, desirable and sustainable.

### Meta-responsibility in ecosystems

This account of responsible ecosystems raises questions about their content and about the location of agency within them. Let us start with the shape of responsibilities within ecosystems. In the previous section, we have already alluded to the network nature of responsibilities in ecosystems. This is easy to see in almost any possible ecosystem of intelligent systems. An example might be the use of intelligent systems for fraud detection in the financial industry. This ecosystem could be delineated by focusing on the financial industry and machine learning approaches to fraud detection based on past cases of identified fraud. An attempt to visualise the networked nature of responsibility relationships in this ecosystem could start with a developer in a software company that produces such systems for the sale to financial institutions. This developer might be responsible for the quality of their work, for adherence to schedules, for cleansing of training data and productive collaboration with colleagues and customers. They might be best placed to understand how their system could disadvantage individuals based on their race or gender and thus be held responsible for minimising biases. The company employing the developer is responsible for adhering to contracts and compliance with the law, for example in data protection. It will be responsible for producing a suitable working environment that allows the developer to discharge their responsibilities. Then there is likely to be a supervisory authority for the financial industry which is responsible for setting expectations and enforcing these, which may include the definition of requirements that the company through the work of the developer has to meet. It is clear that all of these responsibilities interact and form a dense web of responsibilities that can empirically be described in more detail. In addition, the responsibilities are not confined to this particular ecosystem but go far beyond it. The developer may have responsibilities as a parent or a representative of a professional body. The software company is responsible for adhering to standards in other fields of activity beyond financial services and may accept broader corporate social responsibilities. The regulatory authority will be responsible to the government and, by extension, to society. It is likely to have responsibilities to other similar organisations in other jurisdictions. This complexity of intersecting, interacting and overlapping responsibilities is the reason why it makes sense to speak of networks of responsibilities rather than focus on individual responsibility relationships^77^.

The brief example of the network of responsibility in the financial industry for fraud detection could be replicated for other ecosystems of intelligent systems. Such an ecosystem-focused view raises numerous theoretical and practical questions. It would require a detailed understanding of the individual responsibility relationships. Furthermore, there are questions about what characteristics an ecosystem should have, if it is to support the existing network of responsibilities. We use the concept of 'meta-responsibility' to denote a collective view of the responsibilities in an ecosystem. The prefix ‘meta’ (no reference to social media companies intended) is derived from the Greek word-forming element which can mean ‘after, behind’ as well as ‘higher or beyond’^78^. Meta-responsibility thus constitutes a higher level of responsibility, one that does not simply add one more responsibility relationship but aims to cover the responsibility network within the ecosystem. Meta-responsibility has been defined as aiming to “shape, maintain, develop, coordinate and align existing and novel research and innovation-related processes, actors and responsibilities with a view to ensuring desirable and acceptable research outcomes.”^79^.

A responsible ecosystem of intelligent system would thus be one that has successfully established a regime of meta-responsibility which allows existing responsibility relationships to create synergies to ensure that there is answerability for the use of intelligent systems and its consequences. This is a possible answer to the question what a responsible AI ecosystem might be. But it leaves open the question what that would look like in practice. Based on an analysis of the nature of systems in general and ecosystems in particular, one can deduce some requirements that a successful instantiation of meta-responsibility would have to fulfil. Elsewhere we have suggested that an ecosystem that is capable of responding to ethical, social and related concerns would need to have at least three different sets of characteristics^69^. Firstly, it would need to be clearly delineated in terms of time, technology, and geography, to ensure that a specific regime of meta-responsibility could be established. Secondly, it would require a knowledge base that allows its constituent members to discharge their responsibilities. This includes technical knowledge, but also ethical, legal and social knowledge as well as mechanisms to keep this knowledge current. Finally, it would need a governance structure that is adaptive and capable of reacting appropriately to new insights and external influences, e.g. in the form of new technical developments.

Using this responsible ecosystem lens to look at the current AI discourse, one can easily categorise many of the ongoing initiatives and activities as responses to these systems requirements. Many of the detailed research activities, for example around explainable AI or responsibility by design can be understood as part of the knowledge base required by the ecosystem. National and regional legislative and regulatory initiatives form part of the shaping of the governance structure. This is similarly true for corporate governance, e.g. the inclusion of AI into existing risk management or impact assessment structures. One can thus state that the AI discourse appears to promote the move towards responsible ecosystems of intelligent systems. This insight has important implications for scientists and researchers working on intelligent systems as will be spelt out in the conclusion.

## Conclusion

This article argues that the current discourse on AI and ethics, despite its breadth and richness, has structural and fundamental limitations. The discourse provides detailed insights into many of the ethical issues and concerns that AI technologies can raise, and it includes numerous mechanisms that can be employed to address these. One way of assessing the impact of this discourse is to use the concept of responsibility. The current focus of much of the ethics of AI discussion is on identifying specific responsibility relationships based on specific issues, subjects or outcomes. While such individual responsibilities are of crucial importance, they find their limitations in the fact that AI is not so much a clear and well-described technology but can be better described as an ecosystem of socio-technical systems. Based on this conceptualisation of AI the article asked what a responsible ecosystem of intelligent systems would look like and suggested some characteristic that it would have to display.

This argument is important for several audiences. It enriches the theoretical landscape of the discussion of ethics of AI and should thus prove to be of interest to scholars who participate in this discourse. Another audience is made up of scientist and technical experts who work on developing and implementing intelligent systems. Members of this community are generally aware of the ethical and social challenges that intelligent systems can raise. The concept of a responsible ecosystem of intelligent systems can help them think beyond the current sets of responsibilities they are already working with. It can trigger reflections on the delimitation of the ecosystem they work with, on the knowledge that will be required to operate the systems they work on responsibly and on governance mechanisms that may be called for to promote beneficial consequences of the use of these systems.

Overall, the key contribution of the article is thus to offer a novel way of thinking about ethical and social aspects of intelligent systems that is theoretically interesting and practically relevant. Offering a new theoretical perspective does of course not change anything by itself. This article will thus need to trigger a detailed programme of research on the practice of implementing responsible ecosystems using the idea of meta-responsibility. Not all possible delimitations, knowledge provision or governance structures will lead to desired outcomes. In addition, the concept of responsible ecosystems raises new conceptual challenges. One of these is whether such a responsible ecosystem requires its own subject of responsibility, i.e. someone or something that can be held responsible for the consequences of the ecosystem.

The argument put forward in this article is thus no panacea. It does not resolve ethical and social concerns and offers no simple algorithm on how to achieve this. It does, however, offer a new way of thinking about these questions which is fully compatible with the existing discourse, drawing on the now well-established metaphor of the ecosystem. It thus provides a way of thinking about intelligent systems that can help to promote responsibility in AI ecosystems. The consequence should be that ecosystems of intelligent systems will be sensitive to ethical and social questions and promote those technologies and their uses that support human and environmental wellbeing.

## Data Availability

All data generated or analysed during this study are included in this published article.
